# Supplementary material for: 5-Fluorouracil Suppresses Colon Tumor through Activating the p53-Fas Pathway to Sensitize Myeloid-Derived Suppressor Cells to FasL+ Cytotoxic T Lymphocyte Cytotoxicity
Source: Cancers (Basel). 2023 Mar 2;15(5):1563. doi: 10.3390/cancers15051563 (PMC10001142; doi:10.3390/cancers15051563)
Supplement: Supplementary file 1 [file cancers-15-01563-s001.zip › cancers-2244211-supplementary.pdf]

Supplementary Materials: 5-Fluorouracil Suppresses Colon Tumor through Activating the p53-Fas Pathway to Sensitize Myeloid-Derived Suppressor Cells to FasL+ Cytotoxic T Lymphocyte Cytotoxicity

Yingcui Yang, Mingqing Zhang, Yongdan Zhang, Kebin Liu and Chunwan Lu

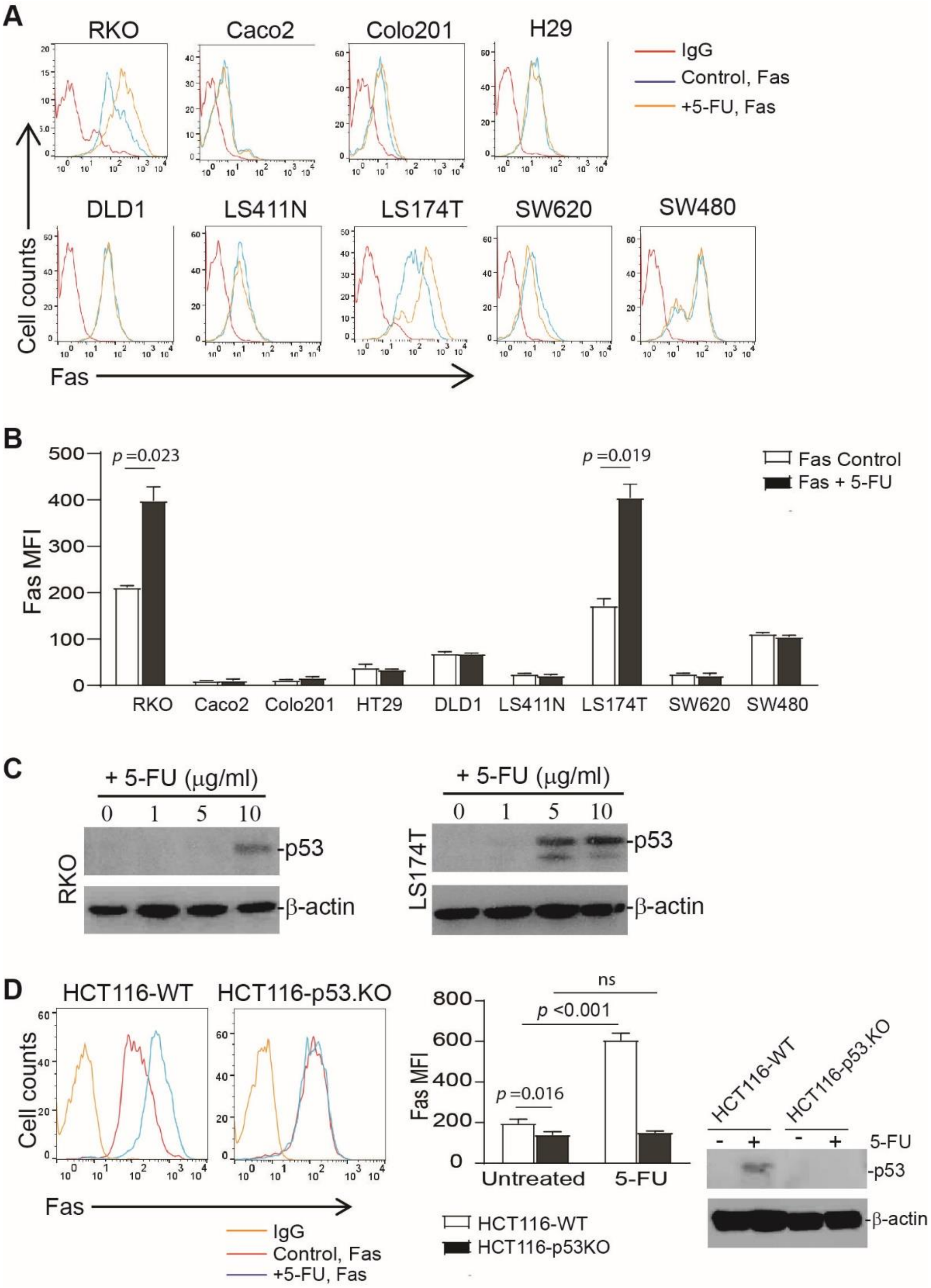

Figure S1. 5-FU activation of Fas expression depends on p53 in human colon tumor cells. **A.** Nine human colon tumor cell lines were cultured in the presence of 5-FU (5 µg/ml) for 24 h and analyzed for Fas expression by flow cytometry. Shown are representative histogram. **B.** Quantification of Fas protein levels (Mean Fluorescent Intensity, MFI) as shown in A. **C.** RKO and LS174T cells were treated with 5-FU at the indicated concentrations for 24 h and analyzed for p53 protein level by Western blotting. **D.** HCT116-WT and HCT116-p53KO cells were treated with 5-FU (5 µg/ml) for 24 h and analyzed for Fas expression by flow cytometry. Shown are representative histogram (left panel) and MFI of Fas protein level (middle panel). This pair of cells were treated with 5-FU (5 µg/ml) for 24 h and analyzed for p53 expression by Western blotting (right panel). The uncropped Western Blot images can be found in Figure S3.

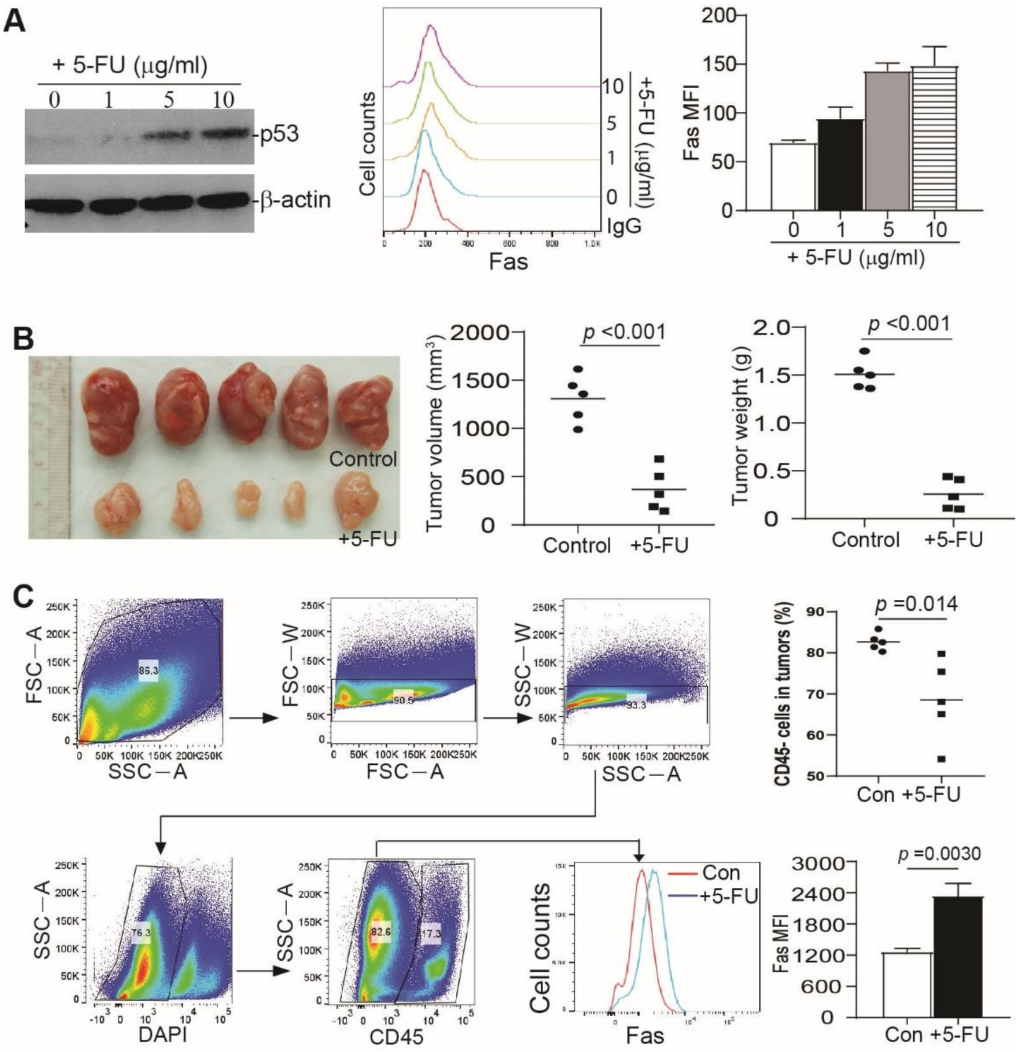

Figure S2. 5-FU induces Fas expression in colon tumor cells in vitro and in vivo. **A.** Left panel: CT26 cells were cultured in the presence of 5-FU at the indicated concentrations for 24h and analyzed for p53 expression by Western blotting. Middle and right panel: CT26 cells were treated with 5-FU at the indicated concentrations for 48 h and analyzed for Fas expression by flow cytometry. Shown is representative histogram (middle panel) and MFI of Fas (right panel). The uncropped Western Blot images can be found in Figure S3. **B.** CT26 cells (2×10^5/mouse) were injected into the right flank of Balb/c mice subcutaneously. Tumor-bearing mice were treated by 5-FU (25 mg/kg body weight) at day 9, once every two days, totally 6 times. Shown on the left is the tumor image, on the right are the tumor volume and weight. **C.** CT26 tumor tissues were digested into single cells and analyzed for Fas expression in $CD45^-$ cells by flow cytometry. Shown on the left is the gating strategy, in the middle is the representative histogram of Fas, on the right is the MFI of Fas and the quantification of $CD45^-$ cells.

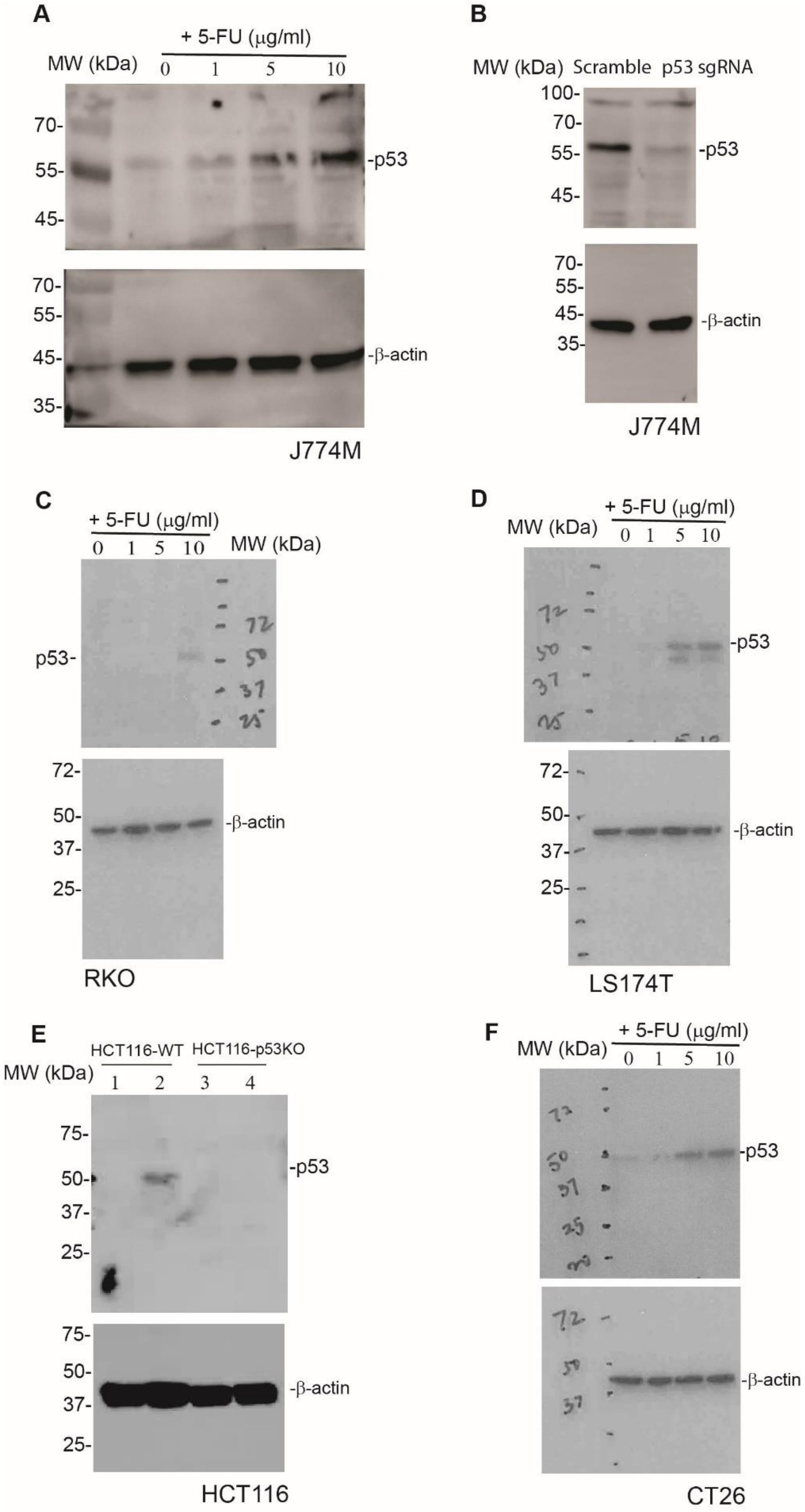

Figure S3. Original Western blots (related to Figures 1A&D, S1C&D and S2A).

Table S1. Information of Patients (related to Figure 7).

Patients	Sex	Age	Tumor Stage	Colon/Rectal Cancer	Chemotherapy
Untreated-1	Female	45	II	Rectal Cancer	No
Untreated-2	Male	74	II	Colon Cancer	No
Untreated-3	Male	67	III	Rectal Cancer	No
Untreated-4	Male	66	I	Colon Cancer	No
Untreated-5	Male	69	II	Rectal Cancer	No
5-FU-1	Male	70	IV	Colon Cancer	mFOLFOX6 + Panitumumab×26
5-FU-2	Female	66	IV	Rectal Cancer	mFOLFOX6 + Panitumumab×15
5-FU-3	Male	67	IV	Colon Cancer	CAPEOX + Bevacizumab×8 + CAPEOX×2
5-FU-4	Male	71	IV	Colon Cancer	mFOLFOX6 + Panitumumab×27

Table S2. TP53 mutations in 10 CRC cell lines.

Cell Line	Mutation
HT29	R273H
DLD1	S241F
CaCo2	E204X
SW480	R273H; P305S
SW620	R273H; P305S
Calo201	Frameshift
LS411N	Y126X
LS174T	WT
HCT116	WT
RKO	WT
